# Staphylococcus aureus Bacteremia in Patients not Meeting Sepsis Criteria: Clinical Features, Host Immune Response and Outcomes

**Published:** 2017-11-20

**Authors:** Daniel E. Salas, Emi Minejima, Joanna Wu, Chong Fang, Joshua Wang, Rosemary She, Paul Nieberg, Annie Wong-Beringer

**Affiliations:** 1 Department of Pharmacy Services, Huntington Hospital, Pasadena, California, United States; 2 School of Pharmacy, University of Southern California, Los Angeles, California, United States; 3 Department of Pathology, Keck Medical Center of USC, Los Angeles, California, United States; 4 Department of Medicine, Huntington Hospital, Pasadena, California, United States

**Keywords:** *Staphylococcus aureus*, Bacteremia, Cytokine, Sepsis

## Abstract

**Background:**

Limitations regarding the sensitivity and specificity of the systemic inflammatory response (SIRS) criteria prompted the recent revision in consensus definitions of sepsis and septic shock. We evaluated patients with *Staphylococcus aureus* bacteremia (SAB) who did not meet SIRS criteria for sepsis (SIRS-negative, SIRS-N) to compare host immune response and outcomes with SIRS-positive (P) patients.

**Methods:**

A prospective observational study of patients hospitalized for SAB during 2012–2015 was conducted. Pro- (TNFα, IL6, IL8) and anti-inflammatory (IL10) cytokine levels (pg/mL) were compared between SIRS-N and SIRS-P patients. Outcome endpoints were day 4 persistence and 30-day mortality.

**Results:**

Of the 353 study patients, 23% were SIRS-N. A similar proportion of SIRS-N and SIRS-P patients had an infection-related admitting diagnosis (70% *vs*. 66%, p=0.5946), and both groups received timely antibiotic administration. Less than 1/3 of SIRS-N group had abnormal WBC count, tachycardia, or tachypnea while <15% had fever/hypothermia or hypotension. Initial proand anti-inflammatory cytokine levels were significantly lower and in balance as indicated by IL10/TNF ratio in SIRS-N compared to SIRS-P patients. IL10/TNF ratio increased progressively in patients with increasing sepsis severity and mortality.

**Conclusions:**

Clinical management of patients with SAB seemed driven largely by clinician assessment rather than SIRS criteria alone, with one in 4 patients not meeting SIRS criteria. Importantly, the severity of presentation and outcomes of SAB correspond well to the magnitude of underlying imbalance in pro- and anti-inflammatory cytokine levels, supporting the updated sepsis definition as “life-threatening organ dysfunction caused by a dysregulated host response to infection”.

**Key points:**

In a prospective observational study of 353 patients with *Staphylococcus aureus* bacteremia, 23% did not meet SIRS criteria for sepsis. Severity of sepsis and risk of death is supported by a dysregulated host cytokine response with progressively increasing IL10/TNF ratio.

## Background

*Staphylococcus aureus* is a predominant cause of bloodstream infections, with an associated mortality of up to 20% [1]. While *S. aureus* is one of the leading organisms implicated in sepsis, reports indicate that only 38% to 44% of patients with *S. aureus* bacteremia (SAB) actually experience severe sepsis or septic shock [2,3]. Previous studies have demonstrated that the presence of severe sepsis/shock is strongly associated with poor outcomes [4], though it is not entirely clear why certain patients progress to this stage of severity in their infection while others do not. The complex host-pathogen interactions and genetic determinants that drive this variable pathophysiologic host response and the associated outcomes have yet to be fully elucidated [5,6]. Furthermore, the degree to which these factors contribute to outcomes among non-septic patients has not been well studied. Kaukonen et al. found that consensus definition of sepsis requiring ≥2 SIRS (systemic inflammatory response syndrome) criteria missed 1 in 8 patients with severe sepsis [7]. Limitations in both the sensitivity and specificity of the SIRS criteria led to revision of the previous sepsis definitions and publication of the updated Third International Consensus Definitions for Sepsis and Septic Shock (Sepsis-3) [8]. The goal of the present study is to apply the previous SIRS-based definition of sepsis to identify the different clinical phenotypes of patients with SAB and relate the varied phenotypes to host cytokine response and outcomes. We hypothesize that patients with SAB who do not meet SIRS criteria (SIRS-N) have overall favorable outcomes compared to SIRS-positive (P) patients, in part due to a less robust but balanced host immune response between pro- and anti-inflammatory cytokines.

## Methods

This was a prospective observational study of patients hospitalized for SAB between July 2012 and June 2015, at three university-affiliated medical centers. This study was approved by the institutional review board at each site. Informed consent was waived as this was an observational study. Patients were eligible for inclusion if they met all of the following: age 18 years, positive blood culture for *S. aureus*, saved bacterial and blood specimens, and receipt of at least 48 hours of effective therapy against *S. aureus*. Those with polymicrobial bacteremia or those who did not receive effective antibiotics within 48 hours of positive blood cultures were excluded from this study. Plasma or serum samples were collected from specimens drawn for routine labs once physician-ordered tests were completed. Samples were collected at onset of SAB and 72 h after start of effective therapy and stored at −80°C until analysis to measure pro-(tissue necrosis factor, TNFα; interleukin-6, IL6; interleukin-8, IL8) and anti-inflammatory (interleukin-10, IL10) cytokine levels (pg/mL) by multiplex Luminex assays. Medical records were reviewed to obtain the following information: demographics, residence prior to admission, comorbid conditions, receipt of immunosuppressive therapy within 30 days of admission which included chemotherapy, monoclonal/polyclonal antibodies (such as rituximab), biologics (such as etanercept), tacrolimus, sirolimus, mycophenolate, or chronic corticosteroids (≥ 20 mg prednisone or equivalent per day); source of bacteremia; clinical presentation (chief complaints on admission, daily vital signs, mental status, need for mechanical ventilation); laboratory values (*i.e*. WBC with differential, days of positive blood culture, culture and sensitivities); echocardiographic findings; details on antimicrobial treatment and surgical interventions; length of hospital stay and survival at 30-day after onset of SAB. Study data were managed using the Research Electronic Data Capture (REDCap) software hosted at the University of Southern California [9].

### Study definitions

SIRS criteria and severity (SIRS-negative, sepsis, severe sepsis, septic shock) were based on vital signs and laboratory values recorded on day 1, in accordance with previous consensus definitions [10]. Sepsis was defined by the presence of at least two or more of the following: temperature >38°C or <36°C, heart rate >90 beats per minute, respiratory rate >20 breaths per minute, and WBC count >12,000/mm^3^ or <4,000/mm^3^ [10]. SIRS-N patients were those who did not meet at least 2 of the above criteria at onset of infection. Severe sepsis included those who met sepsis criteria and had at least one of the following: systolic blood pressure <90 mmHg or a drop ≥40 mmHg, lactate >3 mmol/L, total bilirubin>4 mg/dL, platelet count <100,000/mcL, or PaO_2_/FiO_2_ <300. Septic shock included those who met sepsis criteria and required vasopressor therapy to maintain a mean arterial pressure (MAP) >65. The source of bacteremia was considered “high-risk” if from an endovascular, lower respiratory, intra-abdominal, or central nervous system source, as previously defined by Soriano et al. [11]. “Effective” therapy was defined on the basis of *in vitro* activity against *S. aureus*. Clinical outcome was assessed on day 4 of SAB. Success was defined as either 1) documented or presumed eradication (clinical improvement of signs and symptoms in the absence of repeat blood cultures) or 2) complete resolution or partial improvement of fever, leukocytosis, or signs and symptoms of infection. Failure was defined by any of the following: 1) persistent positive blood culture on day 4, 2) clinical persistence on day 4 (unresolving signs and symptoms of infection or worsening clinical findings), or 3) death.

### Data analysis

Patients were grouped as SIRS-N (negative-less than two) *vs*. SIRS-P (positive - at least two SIRS criteria at infection onset) and by severity of sepsis to compare patient characteristics, host immune response, and clinical outcomes. Host response was analyzed on the basis of cytokine profiles as related to the different clinical phenotypes of patients infected with *S. aureus* bloodstream infection: SIRS-N, SIRS-P (sepsis, severe sepsis, septic shock). Outcome endpoints were clinical response on day 4 after initiation of effective therapy and 30-day mortality.

### Statistical analysis

Categorical variables were compared using Fisher’s exact test. Continuous variables were analyzed using the Mann-Whitney or Kruskall-Wallis test. A p value of <0.05 was considered statistically significant. Graphpad Prism 7 (version 7.0, www.graphpad.com, San Diego, CA) was used for statistical analyses.

## Results

A total of 353 patients with SAB were included in this analysis. Of those, 23% (n=82) were SIRS-N. Among those who were SIRS-P (n=271), 66% had sepsis, 18% severe sepsis, and 16% shock. Baseline demographics were comparable between SIRS-N and SIRS-P patients, though SIRS-N patients were younger (mean age 54 *vs*. 59 years; p=0.0314) and less likely to have a comorbid condition (90%, 74/82 *vs*. 96%, 261/271; p=0.0419) (Table 1).

Hypertension was the most common comorbid condition, accounting for about half of all study patients. Chronic pulmonary disease was the single comorbidity that was significantly lower among SIRS-N patients (3.7%, 3/82 *vs*. 12.5%, 34/271; p=0.0224). Twice as many SIRS-N patients received immunosuppressive therapy at baseline compared to SIRS-P patients (22%, 18/82 *vs*. 10%, 28/271; p=0.0088) (Table 2).

### Clinical presentation and initial management

A similar proportion of SIRS-N and SIRS-P patients were admitted with an infection-related diagnosis (70% *vs*. 66%, p=0.5946). A lower proportion of SIRS-N patients had temperature >38C or <36C (14%, 9/64 *vs*. 55%, 142/257 p<0.0001), heart rate >90 beats/min (29%, 11/38 *vs*. 84%, 113/135 p<0.0001), and respiratory rate >20 breaths/min (26%, 10/38 *vs*. 67%, 90/134 p<0.0001) as well as having WBC >12,000 or <4,000/mm^3^ (30%, 20/67 *vs*. 65%, 179/262 p<0.0001) compared to SIRS-P patients. Methicillin-resistant *S. aureus* (MRSA) was the cause of about one third of all SAB cases in this study with similar proportions between SIRS-N and SIRS-P patients (28%, 23/82 *vs*. 31%, 87/271 p=0.5864). With respect to timing of antibiotic therapy, no significant differences were observed between both groups. The proportion of SIRS-N patients initiated on effective (*in vitro*) therapy on the day of infection onset was 56% (46/82) compared to 65% (175/271) for SIRS-P patients, p=0.1928. Among those patients, the median time to start of effective therapy was 2 hours (interquartile range [IQR] 1–5 hours) *vs*. 1.5 hours (IQR 1–4 hours), p=0.2727, for SIRS-N and SIRS-P patients, respectively. A similar proportion of SIRS-N and SIRS-P patients received consultation by an infectious diseases specialist (52%, 43/82 *vs*. 55%, 150/271). The median time to consultation was delayed by 1 day for SIRS-N compared to SIRS-P patients (3, IQR 1–5 *vs*. 2, IQR 1–3 days, p=0.0231).

### Host cytokine response

**Figure 1:**
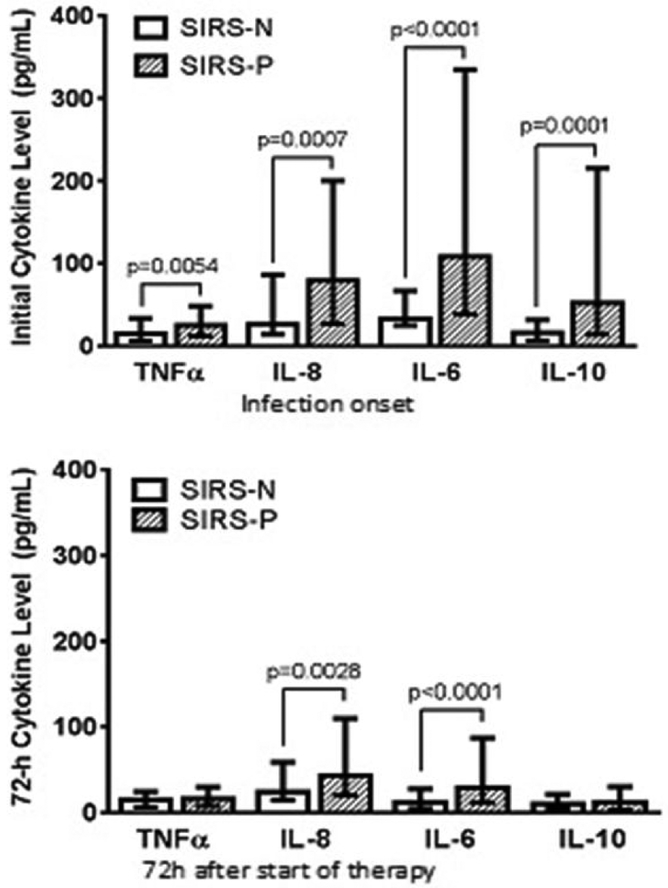
Comparison of Cytokine Response between SIRS-N and SIRS-P patients.

At onset of infection, serum levels of both pro- (TNFα, IL6, IL8) and anti-inflammatory (IL10) cytokines were significantly lower in SIRS-N compared to SIRS-P patients. All cytokine levels decreased following 72 h of effective therapy with similar TNFα and IL10 levels between both groups. However, in SIRS-P patients, IL6 and IL8 levels were significantly more robust at onset and remained significantly elevated at 72 h when compared to SIRS-N patients (Figure 1).

**Figure 2:**
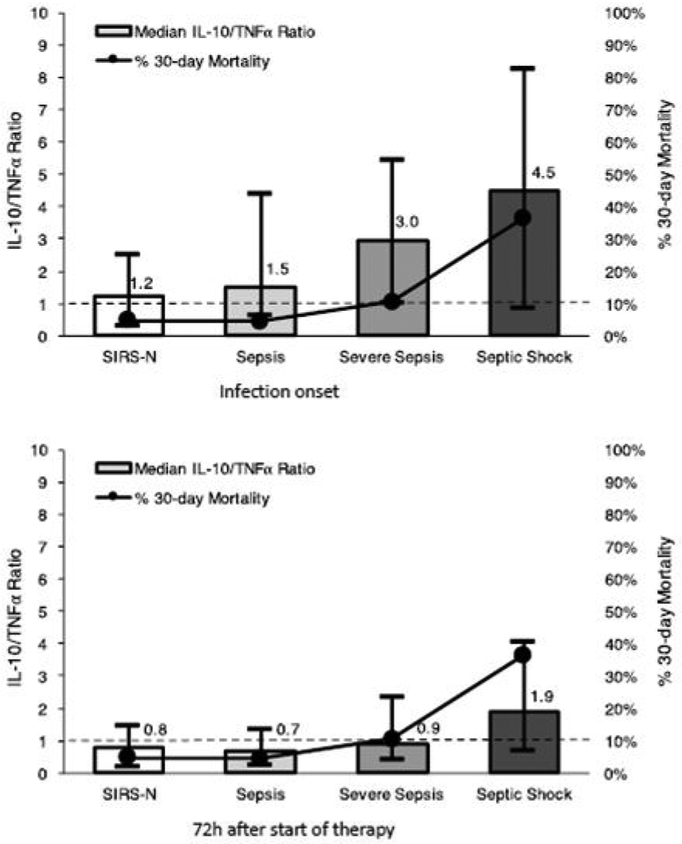
IL10/TNF ratio increases with greater degree of sepsis severity.

To assess the balance between pro- and anti-inflammatory cytokine responses, the ratio of IL10/TNFα was calculated. At onset of infection, SIRS-N patients had the lowest median IL10/TNF ratio (1.2) with progressively increasing value as severity of sepsis increased, at 1.5, 3.0, and 4.5 for patients with sepsis, severe sepsis, and septic shock, respectively (Figure 2). Following 72 h of effective therapy, these ratios normalized to values less than one for all groups except those presenting with septic shock, which remained significantly elevated at 1.9 (Figure 2).

### Outcomes

SIRS-N patients were over 3 times more likely to achieve day 4 clinical success (84% *vs*. 64%, p=0.0001; OR 3.4, 95% CI 1.7–6.7). Only half as many SIRS-N patients required ICU admission at any given time throughout their hospitalization compared to SIRS-P patients (21%, 17/82 *vs*. 42%, 113/271, p=0.0006). A trend toward lower 30-day mortality for SIRS-N patients was also observed (5%, 4/82 *vs*. 11%, 29/271 p=0.1324) (Table 3).

When SIRS-P patients were further grouped by sepsis severity, 30-day mortality rates were 5% (8/179), 10% (5/48), and 36% (16/44), (p<0.0001) for sepsis, severe sepsis, and septic shock patients respectively compared to 5% (4/82) in SIRS-N patients. This is represented graphically in Figure 2 (measured along the secondary Y-axis).

## Discussion

The previous consensus definition for sepsis [10] has been frequently applied in clinical trials of sepsis to enroll eligible patients and in clinical practice to identify patients who need prompt initiation of antibiotic therapy. However, the SIRS-based definition of sepsis has previously been criticized for oversensitivity as a number of non-infectious processes can elicit a SIRS response (*e.g*. trauma, burns) while lacking specificity in some populations since not all patients with an infection experience sepsis [12]. These limitations along with a growing understanding of sepsis pathophysiology eventually led to the updated Sepsis-3 consensus definitions [8]. In our study, we evaluated 353 patients with *S. aureus* bacteremia and found that 23% did not meet the previous sepsis definition threshold of two SIRS criteria at infection onset. Less than 30% of SIRS-N patients exhibited any abnormal values as defined for each physiologic and laboratory parameter used in SIRS criteria for sepsis; white blood cell count, heart rate or respiratory rate were more frequently abnormal than blood pressure or temperature measurements. Regardless of whether SIRS criteria for sepsis were met, up to two-thirds of all study patients were still admitted with an infection-related diagnosis. Importantly, the failure to meet SIRS criteria did not appear to adversely impact the time to initiation of effective antibiotic therapy, a key factor known to affect outcomes in SAB [13,14]. This seems to suggest that clinical care was largely driven by clinician assessment, which may have included a variety of additional parameters beyond those captured by SIRS criteria alone.

As expected, the subtle clinical signs of inflammatory response correlated with a relatively blunted pro- and anti-inflammatory cytokine response at onset in the SIRS-N compared to SIRS-P patients. The pro-inflammatory cytokines selectively measured in our patients (TNF, IL-8, and IL-6) represent some of the key pro-inflammatory mediators involved in what has been referred to as the “cytokine storm”, responsible for the clinical features that characterize sepsis [15]. The difference between SIRS-N and SIRS-P patients was particularly pronounced for IL6 (32.7 *vs*. 108.7 pg/mL, p<0.0001), consistent with previous studies which have highlighted the role of IL6 as an early marker of infection and severity particularly in complicated *S. aureus* bloodstream infection [16,17]. Similarly, the anti-inflammatory cytokine (IL-10) response in SIRS-N patients was also significantly less robust at onset compared to SIRS-P patients (16.3 *vs*. 52.5, p=0.0001). IL10 is responsible for potentiating the compensatory anti-inflammatory environment that has been termed the “immune-paralysis” phase of sepsis which can occur early and simultaneously during the pro-inflammatory cytokine storm [18]. Importantly, we have previously published our findings that an early dysregulated balance between proand anti-inflammatory cytokine response as indicated by an elevated IL10/TNF ratio at 72 h after the start of effective therapy is the strongest predictor for persistence and mortality in SAB [19]. This finding was also supported by others who demonstrated that an elevated IL10/TNFα ratio was predictive of mortality in sepsis [20,21]. As shown in this study cohort, the median IL10/TNF ratio was lowest among SIRS-N patients at 1.2 and increased progressively with increasing severity of sepsis to 4.5 among those with septic shock. The initially elevated IL10/TNF ratio normalized to <1 for both SIRS-N and SIRS-P (sepsis and severe sepsis) patients following 72 h of effective therapy while those with septic shock remained elevated at 1.9. The latter corresponded to a 30-day mortality rate of 36% compared to 5–10% for those with ratio of <1.

## Conclusion

In conclusion, previous SIRS-based definition of sepsis failed to identify 1 in 5 patients with SAB. SIRS-N patients with SAB present with subtle signs of altered physiologic response indicative of a systemic infection, however this does not appear to adversely impact initial clinical assessment and antibiotic management. This altered clinical presentation of SIRS-N patients corresponds to a relatively blunted but balanced pro- and anti-inflammatory cytokine response reflective of immune competence compared to SIRS-P patients whose cytokine profiles were increasingly predominated by anti-inflammatory response suggestive of immunoparalysis as sepsis severity increased. Accordingly, SIRS-N patients experienced more favorable clinical outcomes compared to SIRS-P patients, which was likely due in part to their balanced immune response. Taken together, our findings support the updated Sepsis-3 definition as life-threatening organ dysfunction caused by a dysregulated host response to infection. Furthermore, these findings highlight the potential role for biomarker-directed immune activating therapy as adjunctive treatment for patients with severe/complicated SAB.

## Figures and Tables

**Table 1:** Comparison of baseline characteristics of SIRS-N *vs*. SIRS-P patients with SAB.

Characteristics	SIRS-N (n=82)		SIRS-P (n=271)		P value
**Age (years), mean ± standard deviation**	**54.07**	**± 16.55**	**58.99**	**± 16.61**	**0.0314**
Male	62	75.6%	181	66.8%	0.1372
Residence prior to admission					
Home	63	76.8%	187	69.0%	0.2121
Hospital	9	11.0%	24	8.9%	0.5241
Healthcare-associated facility	6	7.3%	34	12.5%	0.2351
Comorbidity					
**None**	**8**	**9.8%**	**10**	**3.7%**	**0.0419**
1 comorbid condition	22	26.8%	52	19.2%	0.163
≥ 2 comorbid conditions	52	63.4%	209	77.1%	0.0152
Hypertension	37	45.1%	147	54.2%	0.1659
Hyperlipidemia	13	15.9%	59	21.8%	0.2763
Diabetes	31	37.8%	111	41.0%	0.7
Heart failure	8	9.8%	32	11.8%	0.6945
**Chronic pulmonary disease**	**3**	**3.7%**	**34**	**12.5%**	**0.0224**
Cancer	6	7.3%	38	14.0%	0.128
Hepatic impairment	10	12.2%	47	17.3%	0.3075
Renal impairment	25	30.5%	82	30.3%	1.0000
**Immunosuppressive therapy**	**18**	**22.0%**	**28**	**10.3%**	**0.0088**
History of S. *aureus* Infection	18	22.0%	50	18.5%	0.5231

**Table 2:** Clinical presentation and management at infection onset.

	SIRS-N (n = 82)		SIRS-P (n = 271)		P value
Infection-related Admission Diagnosis^a^	57	69.50%	179	66.10%	0.5946
SIRS Criteria at Onset (Day 1)					
Tmax >38 or <36, (%)	12	14.60%	141	52.00%	<0.0001
HR, >90, (%)	25	30.50%	226	83.40%	<0.0001
RR, >20, (%)	17	20.70%	151	55.70%	<0.0001
SBP <90, (%)	7	8.50%	53	19.60%	0.019
WBC >12 or <4, (%)	24	29.30%	183	67.50%	0.0009
Effective therapy started before infection onset	9	11.00%	18	6.60%	0.2341
Effective therapy started day of infection onset	46	56.10%	175	64.60%	0.1928
Time to start of effective therapy, median hours (IQR)	2	(1–5)	1.5	(1–4)	0.2727
Infectious Diseases Consult Obtained	43	52.40%	150	55.40%	0.7045
Days to ID consult, median days (IQR)	3	(1–5)	2	(1–3)	0.0231
Pitt bacteremia score ≥4	2	2.40%	51	18.80%	<0.0001
High-Risk Source of SAB^b^	14	17.10%	68	25.10%	0.139
Primary source of infection^c^					
Line infection	10	12.20%	29	10.70%	0.6905
Endocarditis	3	3.70%	13	4.80%	>0.9999
SSTI	15	18.30%	42	15.50%	0.6074
Osteomyelitis (total)	9	11.00%	22	8.10%	0.5034
Osteomyelitis (non-spinal)	3	3.70%	13	4.80%	>0.9999
Osteomyelitis (spinal)	6	7.30%	9	3.30%	0.1247
Diabetic foot infection	1	1.20%	2	0.70%	0.5487
Surgical wound	3	3.70%	10	3.70%	>0.9999
Pyomyositis	1	1.20%	1	0.40%	0.4111
Non-spinal abscess	3	3.70%	14	5.20%	0.7714
Spinal Abscess	2	2.40%	10	3.70%	0.74
Pneumonia	4	4.90%	26	9.60%	0.2576
Urinary tract infection	4	4.90%	7	2.60%	0.2887
Cardiac device infection	5	6.10%	11	4.10%	0.5431
Non-cardiac device infection	2	2.40%	1	0.40%	0.136
Hardware infection	1	1.20%	10	3.70%	0.4685
Dialysis Catheter infection	10	12.20%	21	7.70%	0.2637
Decubitus ulcer	2	2.40%	7	2.60%	>0.9999
Unknown	17	20.70%	42	15.50%	0.3104

a Infection-related diagnoses included a wide variety of infectious syndromes such as skin and soft tissue infection, pneumonia, urinary tract infection, osteomyelitis, endocarditis, bacteremia, sepsis of unknown etiology, and other (no significant between group differences identified).

b “High-risk Source” includes endovascular, lower respiratory tract, or intra-abdominal source [11].

c Values do not add to 100% as not all sources reported and patient may have had more than one suspected source.

IQR = interquartile range

**Table 3:** Clinical Course and Outcomes.

	SIRS-N (n = 82)		SIRS-P (n = 271)		P value
ICU Admission During Hospitalization	17	20.7%	113	41.7%	0.0006
Day 4 success	71	86.6%	178	65.7%	0.0002
Length of stay, median days (IQR)	10.5	(6, 18)	11	(7, 22)	03186
30-day mortality	4	4.9%	29	10.7%	0.1324
